# Investigation of Attenuation Correction Methods for Dual-Gated Single Photon Emission Computed Tomography (DG-SPECT)

**DOI:** 10.3390/bioengineering12111195

**Published:** 2025-11-01

**Authors:** Noor M. Rasel, Christina Xing, Shiwei Zhou, Yongyi Yang, Michael A. King, Mingwu Jin

**Affiliations:** 1 Department of Physics, University of Texas at Arlington, Arlington, TX 76019, USA; nxr7226@mavs.uta.edu (N.M.R.); christina.xing@mavs.uta.edu (C.X.); shiwei.zhou@mavs.uta.edu (S.Z.); 2 Department of Electrical and Computer Engineering, Illinois Institute of Technology, 3301 S Dearborn St., Chicago, IL 60616, USA; yangyo@illinoistech.edu; 3 Department of Radiology, University of Massachusetts Chan Medical School, Worcester, MA 01655, USA; michael.king@umassmed.edu

**Keywords:** cardiac-respiratory dual-gated SPECT (DG-SPECT), attenuation correction, motion match, 4D reconstruction

## Abstract

Background: Cardiac-respiratory dual gating in SPECT (DG-SPECT) is an emergent technique for alleviating motion blurring artifacts in myocardial perfusion imaging (MPI) due to both cardiac and respiratory motions. Moreover, the attenuation artifact may arise from the spatial mismatch between the sequential SPECT and CT attenuation scans due to the dual gating of SPECT data and non-gating CT images. Objectives: This study adapts a four-dimensional (4D) cardiac SPECT reconstruction with post-reconstruction respiratory motion correction (4D-RMC) for dual-gated SPECT. In theory, a respiratory motion-matched attenuation correction (MAC) method is expected to yield more accurate reconstruction results than the conventional motion-averaged attenuation correction (AAC) method. However, its potential benefit is not clear in the presence of practical imaging artifacts in DG-SPECT. In this study, we aim to quantitatively investigate these two attenuation methods for SPECT MPI: 4D-RMC (MAC) and 4D-RMC (AAC). Methods: DG-SPECT imaging (eight cardiac gates and eight respiratory gates) of the NCAT phantom was simulated using SIMIND Monte Carlo simulation, with a lesion (20% reduction in uptake) introduced at four different locations of the left ventricular wall: anterior, lateral, septal, and inferior. For each respiratory gate, a joint cardiac motion-compensated 4D reconstruction was used. Then, the respiratory motion was estimated for post-reconstruction respiratory motion-compensated smoothing for all respiratory gates. The attenuation map averaged over eight respiratory gates was used for each respiratory gate in 4D-RMC (AAC) and the matched attenuation map was used for each respiratory gate in 4D-RMC (MAC). The relative root mean squared error (RMSE), structural similarity index measurement (SSIM), and a Channelized Hotelling Observer (CHO) study were employed to quantitatively evaluate different reconstruction and attenuation correction strategies. Results: Our results show that the 4D-RMC (MAC) method improves the average relative RMSE by as high as 5.42% and the average SSIM value by as high as 1.28% compared to the 4D-RMC (AAC) method. Compared to traditional 4D reconstruction without RMC (“4D (MAC)”), these metrics were improved by as high as 11.23% and 27.96%, respectively. The 4D-RMC methods outperformed 4D (without RMC) on the CHO study with the largest improvement for the anterior lesion. However, the image intensity profiles, the CHO assessment, and reconstruction images are very similar between 4D-RMC (MAC) and 4D-RMC (AAC). Conclusions: Our results indicate that the improvement of 4D-RMC (MAC) over 4D-RMC (AAC) is marginal in terms of lesion detectability and visual quality, which may be attributed to the simple NCAT phantom simulation, but otherwise suggest that AAC may be sufficient for clinical use. However, further evaluation of the MAC technique using more physiologically realistic digital phantoms that incorporate diverse patient anatomies and irregular respiratory motion is warranted to determine its potential clinical advantages for specific patient populations undergoing dual-gated SPECT myocardial perfusion imaging.

## 1. Introduction

Single Photon Emission Computed Tomography (SPECT) Myocardial Perfusion Imaging (MPI) is a popular and cost-effective noninvasive imaging modality in clinical nuclear medicine. It provides physiological information about the heart muscle and is used to assess various cardiac conditions, including hypertrophic cardiomyopathy and coronary artery disease (CAD) [1,2,3]. However, the quality of SPECT MPI images often declines due to a range of degrading factors, such as reduced image contrast caused by scatter, artifacts from attenuation, distance-dependent blurring, and motion artifacts arising from both voluntary and involuntary patient motion [4,5]. Cardiac and respiratory motions are among the most significant sources of image degradation in nuclear cardiology. Reference [6] comprehensively reviewed motion types and correction strategies, emphasizing that these physiological motions are major contributors to diagnostic inaccuracies in SPECT and PET MPI. Cardiac motion can cause blurring of myocardial perfusion images and apparent wall thickening, which can be mitigated by synchronizing data acquisition with the electrocardiogram (ECG) signal—commonly known as cardiac-gated SPECT (CG-SPECT). While CG-SPECT provides valuable functional parameters such as ejection fraction and wall motion, it often suffers from increased noise because projection counts are distributed over multiple gates (typically 8–16) per cardiac cycle.

Although cardiac gating reduces motion blurring, it does not address respiratory motion, which introduces additional artifacts—particularly in the anterior and inferior walls—leading to an underestimation of perfusion and reduced contrast of small defects [7]. It was also demonstrated that both attenuation and respiratory motion produce significant artifacts in SPECT MPI, highlighting the importance of motion correction for accurate defect detection [8]. Respiratory gating, achieved by either phase gating or amplitude gating, enables image acquisition during motion-minimized portions of the breathing cycle, thereby reducing respiratory artifacts [9]. Extending this concept, it was reported that dual cardiac–respiratory gating can effectively reduce respiratory motion-induced artifacts in SPECT MPI, improving left ventricular shape and wall thickening quantification without compromising image quality [10]. However, dual cardiac–respiratory gated SPECT (DG-SPECT) remains challenging because subdividing projections across both cardiac and respiratory gates greatly increases noise, limiting its clinical adoption [9,11].

While the gating technique mitigates motion artifacts, it also amplifies image noise, prompting the development of advanced four-dimensional (4D) reconstruction methods. Such spatiotemporal algorithms jointly exploit correlations among gated frames to improve reconstruction accuracy and defect detectability [4,12]. The motion-compensated 4D reconstruction strategy has demonstrated significant improvement in both quantitative accuracy and lesion detectability. A fully 4D joint reconstruction framework that simultaneously accounts for both cardiac and respiratory motion in SPECT was proposed and demonstrated significant improvements in image quality and quantitative accuracy compared with conventional gated methods [13]. More recently, a data-driven respiration-gated SPECT approach capable of extracting respiratory signals directly from list-mode data without external sensors was found to improve quantification accuracy over conventional 3D SPECT [14]. Incorporating both cardiac deformation and respiratory motion within 4D SPECT reconstruction frameworks further enhances myocardial wall delineation and quantitative accuracy by reducing motion-related inconsistencies during continuous image acquisition [15].

Despite these algorithmic advances, accurate attenuation correction (AC) remains a critical challenge, particularly for DG-SPECT. AC errors often arise when respiratory motion causes a misalignment between SPECT emission data and CT-derived attenuation maps. Some systematical studies show that even a 1-pixel misregistration between CT and SPECT images can significantly alter regional uptake in MPI, emphasizing the critical need for respiratory motion-matched attenuation correction to ensure accurate myocardial activity estimation [16,17]. Another study showed that both cine average CT (CACT) and interpolated average CT (IACT) effectively reduce respiratory misalignment artifacts and improve quantitative accuracy in myocardial perfusion SPECT/CT compared with conventional helical CT attenuation correction while minimizing radiation dose [18]. Dedicated cardiac SPECT scanners provide higher sensitivity and may enable data-driven dual gating [19]. However, most clinical systems remain based on conventional Anger cameras requiring external motion monitoring. The superior noise-suppression capabilities of advanced 4D reconstruction algorithms have renewed interest in DG-SPECT MPI using these systems [20]. Typically, SPECT data are acquired in list-mode and can be synchronized with both cardiac (through ECG signal) and respiratory motion (through either external monitoring or data-driven methods [19]). However, attenuation maps derived from low-dose cone-beam CT (CBCT) are often static and ungated. While cardiac motion has a relatively small effect due to the limited displacement of the heart, respiratory motion can introduce substantial mismatch between SPECT and CT datasets, leading to residual attenuation artifacts [21].

Several studies have investigated the impact of attenuation maps on respiratory-gated SPECT MPI. Ref. [22] demonstrated that the attenuation map from a slow cone-beam CT (CBCT) resulted in a better uniform heart wall in SPECT images after attenuation correction than the fast diagnostic helical CT. In phantom studies, refs. [23,24] showed that interpolated and respiratory-gated CT attenuation maps yielded the smallest relative differences in polar maps when compared with dual-gated CT used as the reference standard for DG-SPECT attenuation correction. Theoretically, a respiratory motion-matched attenuation correction (MAC) approach should yield more accurate reconstructions than the conventional motion-averaged attenuation correction (AAC) method. However, its potential benefit is not clear in the presence of practical imaging artifacts in DG-SPECT, where noise, misregistration, and limited photon counts can obscure potential improvements. In this study, we quantitatively investigate the effects of different attenuation maps used for DG-SPECT AC within a joint 4D reconstruction framework. Specifically, 4D reconstruction is performed for DG-SPECT in each respiratory gate using either the motion-matched attenuation correction (MAC) or motion-averaged attenuation correction (AAC) approach. A post-reconstruction respiratory motion-compensated smoothing is then applied to further suppress the elevated noise. In addition to conventional quantitative metrics for SPECT reconstruction quality, such as relative root mean squared error (RMSE) and Structural Similarity Index Measurement (SSIM), we also employ a Channelized Hotelling Observer (CHO) study to evaluate cardiac defect (lesion) detectability at different locations.

## 2. Methods

### 2.1. DG-SPECT Imaging Model

For dual-gated SPECT (DG-SPECT) imaging, the projections are synchronized with both the respiratory and cardiac cycles. The projection data are described by the following equation:

(1) $$E\left[P_{r,k}\right]=HI_{r,k}+s_{r,k};r=1,\;\ldots ,\;R,\;k=1,\;\ldots ,\;K.\;$$

where $P_{r,k}$, $I_{r,k},\;$ and $s_{r,k}$ represent the projection (sinogram) data, original image data, and expected scatter component for the respiratory bin r and cardiac gate k. $E\left[\cdot \right]$ is the expectation operator. K and R are the total number of gates (note that we use “gate”, “bin”, and “phase” interchangeably in the following text) for both respiratory and cardiac motion. H is the system matrix for the projection operation, where the depth-dependent blur is always included [25], while the attenuation effects are included using either a respiratory motion-matched attenuation map for MAC or an averaged attenuation map for AAC.

When solely considering cardiac motion in CG-SPECT, a relatively large number of photons are available for each cardiac phase, i.e., all *R* respiratory phases can be utilized for each *k*. On the other hand, if both cardiac and respiratory motions are considered in DG-SPECT, the photon count diminishes significantly, as only one respiratory phase *r* per cardiac gate ***k*** will be treated as motionless. Let each DG-SPECT projection be represented as:

$$P_{r=1,k=1},P_{r=2,\;k=1},{\ldots ,P}_{r=R,k=1},P_{r=1,k=2},P_{r=2,k=2},{\ldots ,P}_{r=R,k=2},\ldots ,P_{r=1,k=K},P_{r=2,k=K},{\ldots ,P}_{r=R,\;k=K}.$$

Then, each CG-SPECT projection would be $P_{k=1}=P_{r=1,k=1}+P_{r=2,k=1}+{\ldots +P}_{r=R,k=1}$, using cardiac gate one as an example.

To investigate the respiratory motion-matched attenuation correction (MAC) for DG-SPECT, for each respiratory phase r, H includes an attenuation map ***Att_r*** corresponding to the current respiratory phase. While investigating the respiratory motion-averaged attenuation correction (AAC), H only includes the same attenuation map ***Att_avg*** for all the respiratory phases.

### 2.2. 4D Reconstruction for DG-SPECT for Each Respiratory Phase Independently

**Figure 1:**
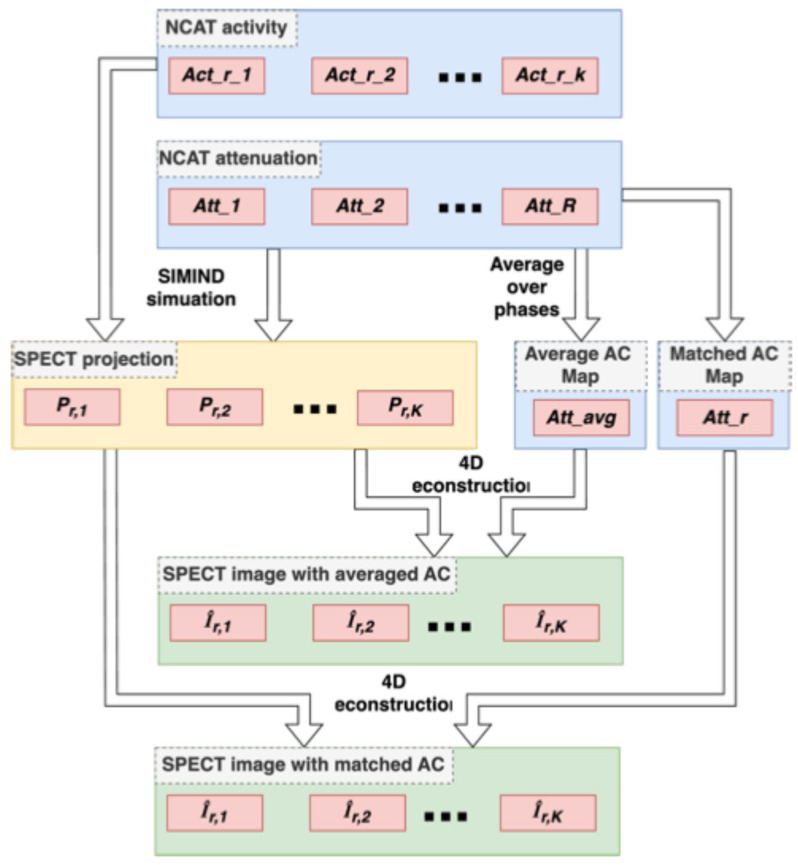
Four-dimensional reconstruction of DG-SPECT image sequences for *r*^th^ respiratory phase with matched attenuation correction (MAC) and averaged attenuation correction (AAC). “*Act_r*_#” represents the activity map for the *r*^th^ respiratory phase and the #^th^ cardiac phase; “*Att_r*” represents the attenuation map for the *r*^th^ respiratory phase.

In the DG-SPECT study, we initially reconstructed all cardiac-gated images for each respiratory phase independently by utilizing projections from all cardiac gates. The primary distinction lies in whether the respiratory motion-averaged attenuation map (***Att_avg***) or the matched attenuation map (***Att_r***) is employed for the system matrix corresponding to respiratory phase *r*. For simplicity, we set the respiratory phase *r* **=** 1. Let ***P***_1_ represent the projection data across all cardiac gates, i.e., $P_{1}\equiv [P_{1,1},P_{1,2},\ldots ,P_{1,K}]$, and let $I_{1}\equiv [I_{1,1},I_{1,2},\ldots ,I_{1,K}]$ be the associated cardiac-gated images for respiratory phase 1. Subsequently, the cardiac motion-compensated four-dimensional (4D) reconstruction can be realized through the *maximum a posteriori* (MAP) estimation as [4].

(2) $${\hat{I}}_{1}=\arg\underset{I_{1}}{\max}\left\{logp\left(P_{1}|I_{1}\right)+logp(I_{1})\right\}$$

where $p\left(P_{1}|I_{1}\right)$**,** the likelihood function of ***P***_1_ is parameterized by ***I***_1_, and $p(I_{1})$ is the prior on ***I***_1_. The ***p***(***I***_1_) prior distribution is used to alleviate the noise effect in the reconstructed images and is defined in the form of a separable Gibbs prior as follows:

(3) $$p\left(I_{1}\right)\propto \exp\left[-\beta_{s}U_{s}\left(I_{1}\right)-\beta_{t}U_{t}\left(I_{1}\right)\right],$$

where $U_{s}\left(I_{1}\right)$ is an energy term defined to enforce spatial smoothing within individual cardiac gates, $U_{t}\left(I_{1}\right)$ is an energy term to enforce smoothing along the motion trajectories across the different cardiac gates, and $\beta_{s}$ and $\beta_{t}$ correspond to the scalar weighting factors called spatial and temporal smoothing parameters. In this study, we applied the modified block sequential regularized expectation-maximization (BSREM) algorithm to obtain the solution of Equation (2), as in our previous works [4]. The 4D reconstruction with MAC and AAC for *r*^th^ phase is shown in Figure 1.

As a reference for quantitative evaluation, we reconstructed the ground truth images using the Ordered-Subset Expectation Maximization (OSEM) reconstruction algorithm (10 iterations and 16 subsets) on the noise-free projection data by turning off attenuation and scatter in the SIMIND simulation and reconstruction process. However, the depth-dependent blurring was included in both simulation and reconstruction processes in obtaining ground truth images. We refer these ground truth images as the “ideal image” in the following text unless otherwise stated.

Using a set of $\beta_{s}$ and $\beta_{t}$, we obtained a noisy 4D reconstruction for a reference respiratory phase (phase 1) and then computed the root mean squared error (RMSE) between the noisy reconstruction images and ideal images. The optimal $\beta_{s}$ and $\beta_{t}$ values were chosen based on the lowest RMSE values.

### 2.3. Post-4D Reconstruction Respiratory-Motion Compensated Smoothing for DG-SPECT

We applied 4D reconstruction for *r* = 1, 2, …, *R* to obtain images for all respiratory phases, as shown in Figure 1. Since the photon count of each dual-gated bin is very low, we summed images over all *k* cardiac phases to alleviate the photon noise for the respiratory motion estimation, as shown in Equation (4), where ***r*** denotes the *r^th^* respiratory phase and *k* denotes the *k^th^* cardiac gate,

(4) $${Img}_{r}=\sum_{k=1}^{K}{\hat{I}}_{r,k}\;for\;r=1,\;2,\;\ldots ,\;R.$$

The summed images are used to estimate the respiratory motion. The deformation vector field (DVF) from respiratory phase *i* to respiratory phase *j* can be estimated as Equation (5) [26]:

(5) $${DVF}_{j\leftarrow i}=\frac{(Img_{j}-Img_{i})\vec{\nabla }Img_{i}}{{\left\|\vec{\nabla }Img_{i}\right\|}^{2}+{\left\|Img_{j}-Img_{i}\right\|}^{2}},\;for\;i=1,2,\ldots R\;and\;j=1,2,\ldots ,R.$$

When *i* = ***j***, the DVF is zero. Based on the DVF obtained from Equation (5), we can obtain a warped image, ${\tilde{I}}_{j\leftarrow i,k}$ at respiratory phase *j* from the image ${\hat{I}}_{i,k}$ at respiratory phase ***i*** for a fixed cardiac phase ***k*** as

(6) $${\tilde{I}}_{j\leftarrow i,k}=Imwarp\left({\hat{I}}_{i,k},{DVF}_{j\leftarrow i}\right)$$

Thus, the image at respiratory phase ***j*** and cardiac phase *k* can be denoised by a weighted sum of images from all respiratory phases. Since the estimated DVFs are more accurate for the adjacent phases, we use the following weighted sum to obtain a final temporally filtered image $I_{j,k}^{*}$:

(7) $$I_{j,k}^{*}=\sum_{i=1}^{R}\left|1-2\cdot \frac{\left|i-j\right|}{R}\right|\cdot {\tilde{I}}_{j\leftarrow i,k}$$

**Figure 2:**
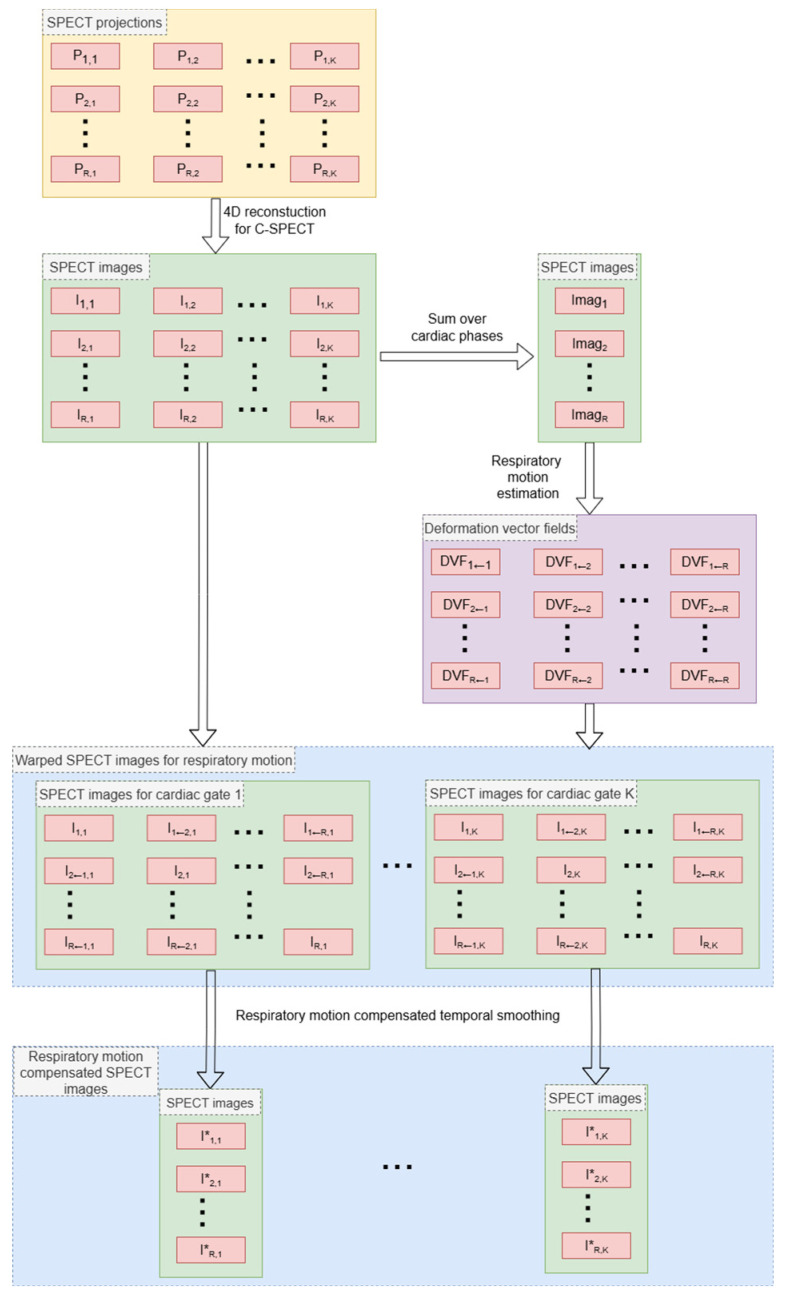
4D reconstruction with post-reconstruction respiratory motion-compensated smoothing for DG-SPECT that summarizes Equations (1)–(7).

The whole workflow of 4D DG-SPECT reconstruction with motion-compensated post-reconstruction smoothing is shown in Figure 2 that summarizes Equations (1)–(7).

## 3. Experiments

### 3.1. Phantoms and DG-SPECT Monte Carlo Simulation

**Figure 3:**
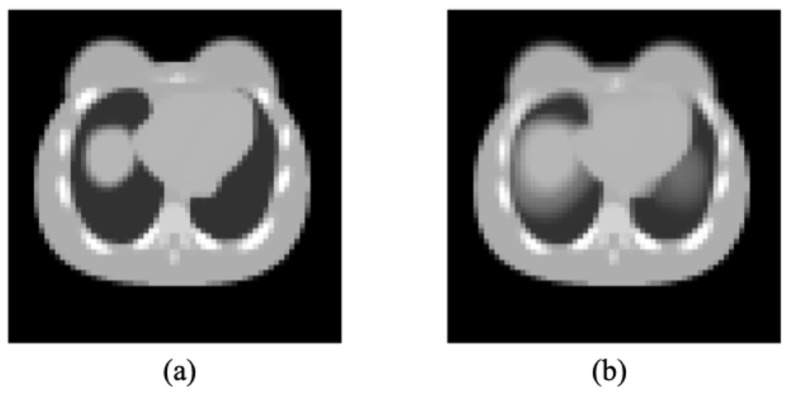
(**a**) A respiratory motion-matched attenuation map (phase 4) and (**b**) motion-averaged attenuation map for the NCAT female phantom.

The 4D NURBS-based cardiac-torso (NCAT) 2.0 female phantom was used to generate activity (source distribution) and attenuation maps by incorporating cardiac motion (8 gates) and respiratory motion (8 gates) for dual-gated SPECT imaging. The female phantom had the following anatomical dimensions: a body long axis of 33 cm, a body short axis of 25 cm, a rib long axis of 28 cm, and a rib short axis of 21 cm. The breast dimensions included a long axis of 18.2 cm, a short axis of 7 cm, and a height of 14 cm. The extent of superior and inferior motion of the diaphragm was 2 cm, while the anterior–posterior expansion of the chest was 1.2 cm in the body-axis, and the lateral motion was minimal. These parameters were set to simulate normal breathing within the NCAT software. The corresponding motion of the heart was calculated to be 1.59 cm along the superior–inferior axis and 0.95 cm along the anterior–posterior axis, as defined in NCAT. The respiratory period and cardiac cycle were 5 s and 1 s, respectively. Figure 3 shows the respiratory motion-matched attenuation map (using respiratory phase 4 as an example) and the motion-averaged attenuation map generated by the NCAT 2.0 software in this study.

The SIMIND Monte Carlo simulation tool [27] was employed to simulate the DG-SPECT myocardial perfusion imaging using ^99m^Tc-Sestamibi as the imaging agent. The SPECT system simulated was the Philips Prism 3000 (Philips Medical Systems, Inc., Cleveland, Ohio, USA) equipped with a low-energy high-resolution (LEHR) collimator. The projection matrix consisted of 64 × 64 pixels, with a pixel size of 0.634 cm. For a circular camera rotation with a radius of 28.5 cm, 64 projections evenly distributed over 360° were collected for each cardiac and respiratory gate. The simulation modeled distance-dependent blurring, attenuation, and scatter, except for the ideal reconstruction, where attenuation and scatter were not included. For the simulation, the average spatial resolution at the heart location within the image slices was approximately 1.3 cm in full width at half maximum (FWHM). A total of 8 million counts (for all organs in the field of view) typical in cardiac SPECT imaging studies were used to generate Poisson noise, which led to an average of 1 × 10^6^ photons per respiratory gate (with 8 cardiac gates) or an average of 1.25 × 10^5^ photons per respiratory and cardiac phase.

To assess the performance of lesion detectability, a transmural perfusion defect (lesion) was introduced to the left ventricular myocardium with a 20% reduction in ^99m^Tc-Sestamibi uptake and a size of 20 mm along the long-axis dimension, and a circumferential extent of 100 degrees. The lesion was moved with the heart and lung motion. To examine the influence of lesion locations, perfusion defects were introduced at four distinct locations: anterior, inferior, lateral, and septal.

### 3.2. Reconstruction and Attenuation Correction Methods for Comparison and Evaluation Criteria

#### 3.2.1. Reconstruction and Attenuation Correction Methods

We proposed three methods for DG-SPECT: (1) a 4D DG-SPECT reconstruction with the respiratory motion-compensated post-filtering and respiratory motion-matched attenuation correction 4D-RMC (MAC), (2) a 4D DG-SPECT reconstruction with the respiratory motion-compensated post-filtering and respiratory motion-averaged attenuation correction 4D-RMC (AAC), and (3) the 4D CG-SPECT reconstruction using respiratory motion-matched attenuation correction, but without respiratory motion-compensated post-recon filtering, 4D (MAC). The last is to check whether the respiratory motion-compensated post-filtering improves 4D cardiac reconstructions for dual-gated SPECT. Since 4D (AAC) behaves similar to 4D (MAC), it is omitted in the following analysis. For simplicity, scatter correction is also omitted from reconstruction.

#### 3.2.2. Evaluation Criteria

1. Quantitative accuracy of the reconstructed myocardium: To quantify the overall accuracy of reconstructed myocardium, the relative root mean squared error (RMSE) and structural similarity index measurement (SSIM) are calculated for the reconstructed images of a 28 × 28 × 10 volumetric region for the majority of LV. The mean relative RMSE and mean SSIM indices are computed for all cardiac gates within the corresponding respiratory gate. The ideal images described in Section 2.2 are used as the ground truth for the calculation of the relative RMSE and SSIM.
2. Intensity Profile: Moreover, the differences among different reconstruction and AC methods are evaluated by image intensity line profiles across the LV wall in transverse slices.
3. Perfusion defect detection: To quantify the detectability of the introduced perfusion defect in reconstructed images, a Channelized Hotelling Observer (CHO) is employed. In this study, the 28 × 28 LV region in a transverse slice is bilinearly interpolated to a 140 × 140 image. Four rotationally symmetric, non-overlapping input channels are used for the CHO, and internal noise is included as in [4]. A total of 60 noise realizations (30 lesion-present and 30 lesion-absent) are used for each lesion location. The detection performance is summarized by the area under the receiver operating characteristic (ROC) curve (“Az”). This CHO study represented a “signal-known exactly” and “background-known exactly” observer study. The end-diastole (ED) cardiac gate of a reference respiratory gate during which the heart wall is most expanded, is used for the CHO study. We further performed a statistical analysis on the Az of the CHO using MATLAB’s (version R2025a) rocmetrics function. Statistical significance of Az differences was evaluated using nonparametric bootstrap resampling (for 2000 iterations). Two-tailed *p*-values were derived for the null hypothesis for which there was no difference for the Az’s from the two methods. *p* < 0.05 was set as the significance threshold to reject the null hypothesis.

## 4. Results

### 4.1. Quantitative Accuracy of Myocardium Reconstruction

**Figure 4:**
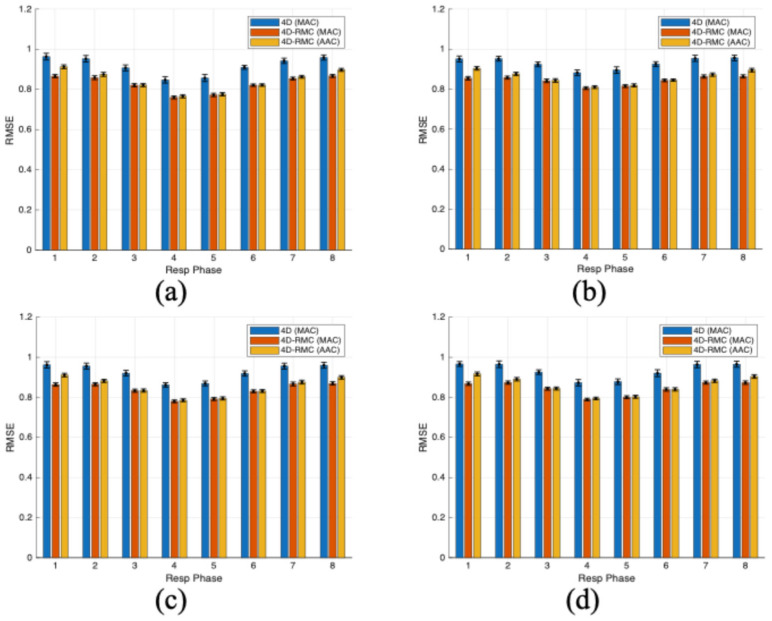
Relative RMSE values across eight respiratory phases from different methods (blue bar: 4D (MAC); red bar: 4D-RMC (MAC); yellow bar: 4D-RMC (AAC)) for four defect locations: (**a**) anterior, (**b**) inferior, (**c**) lateral, and (**d**) septal.

The relative RMSE results of the LV myocardium for four lesion cases reconstructed by different reconstruction methods are summarized in Figure 4. Each bar represents the mean value of eight cardiac gates for each respiratory gate, which was further averaged over 30 noise realizations, and the error bars represent the standard deviation. As can be seen, the blue bars (4D (MAC)) are much higher than the red (4D-RMC (MAC)) and yellow (4D-RMC (AAC)) bars, i.e., the post-reconstruction motion-compensated filtering effectively suppressed noise for lower relative RMSE values. The difference between 4D-RMC (MAC) (red bars) and 4D-RMC (AAC) (yellow bars) is small, although the former is slightly lower than the latter, particularly for the first and last respiratory gates. The biggest improvement was in respiratory phase 1 of the anterior lesion, where the 4D-RMC (MAC) improves the relative RMSE values by 5.42% compared to 4D-RMC (AAC) and by 11.23% compared to 4D (MAC). However, in the middle respiratory phases, the improvement of the MAC over the AAC diminishes.

**Figure 5:**
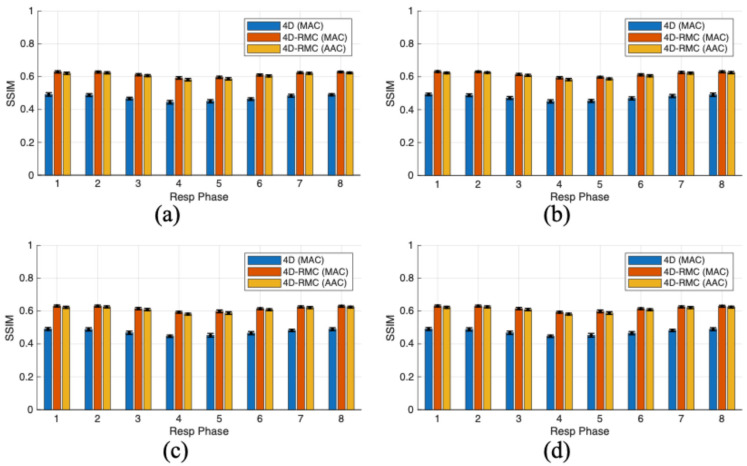
SSIM values across eight respiratory gates from different methods (blue bar: 4D (MAC); red bar: 4D-RMC (MAC); yellow bar: 4D-RMC (AAC)) for four defect locations: (**a**) anterior, (**b**) inferior, (**c**) lateral, and (**d**) septal.

For further quantitative accuracy of LV myocardium for four lesion cases reconstructed by different methods, we plotted the SSIM values shown in Figure 5. Each bar represents the mean value of eight cardiac gates for each respiratory gate, which was further averaged over 30 noise realizations, and the error bars represent the standard deviation. Again, 4D-RMC (MAC) (red bars) and 4D-RMC (AAC) (yellow bars) achieved much higher SSIM values than 4D (MAC), which demonstrates the importance of respiratory motion-compensated denoising. The 4D-RMC (MAC) performs slightly better than the 4D-RMC (AAC). Specifically, the SSIM value of 4D-RMC (MAC) improves by 1.28% compared to 4D-RMC (AAC) and by 27.96% compared to the 4D-MAC method in the first respiratory phase for the anterior lesion case, which was the biggest improvement. Moreover, from Figure 4 and Figure 5, we observed that the variations in relative RMSE and SSIM with different reconstruction methods were almost similar for different lesion locations. This is no surprise as relative RMSE and SSIM are global metrics for image quality.

### 4.2. Intensity Profiles

**Figure 6:**
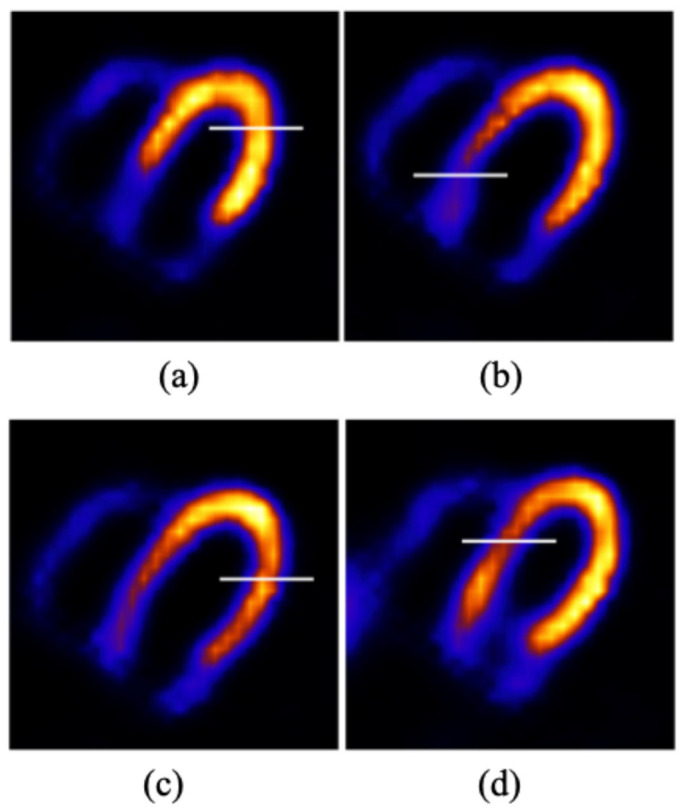
The lines for the intensity profile across the LV wall (where the lesion is located) in reconstructed transverse slices of the ideal images for four lesion locations: (**a**) anterior, (**b**) inferior, (**c**) lateral, and (**d**) septal.

To obtain the image intensity profile, we have drawn a horizontal line across the LV wall in the location of the perfusion defect (lesion) in reconstructed transverse slices. The corresponding lines are presented in the ideal images in Figure 6 for different lesion slices: (a) anterior; (b) inferior; (c) lateral; and (d) septal.

**Figure 7:**
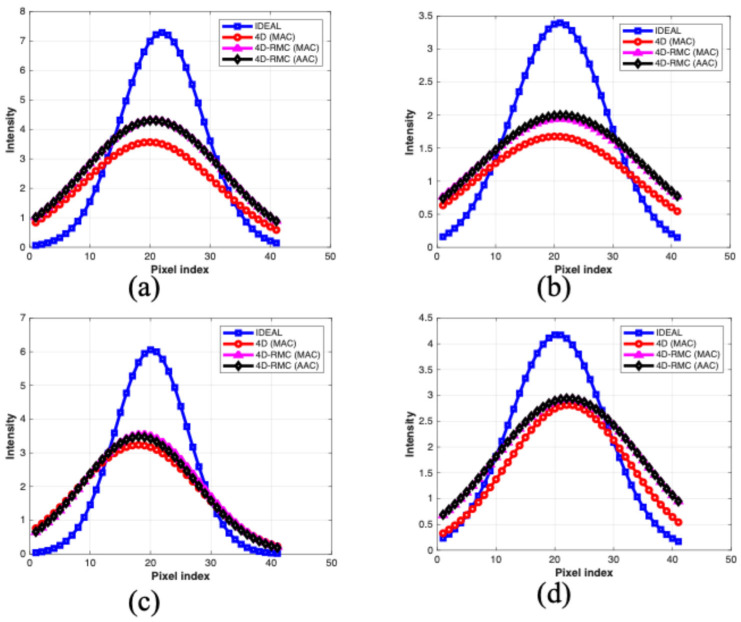
Intensity profiles across the LV wall (where the lesion is located) in reconstructed transverse slices for different methods for four lesion locations: (**a**) anterior, (**b**) inferior, (**c**) lateral, and (**d**) septal. The profiles from the ideal images serve as a reference.

From Figure 7, we observed that the intensity profiles of the 4D-RMC with either the MAC or AAC methods are closer to the ideal profile than that of 4D (MAC). However, a significant difference between the 4D-RMC (MAC) and 4D-RMC (AAC) methods was not observed. The average AC seems to be marginally better than the matched AC for the inferior lesion, while the reversed relation exists for the lateral lesion.

### 4.3. Perfusion Defect Detection Performance

**Figure 8:**
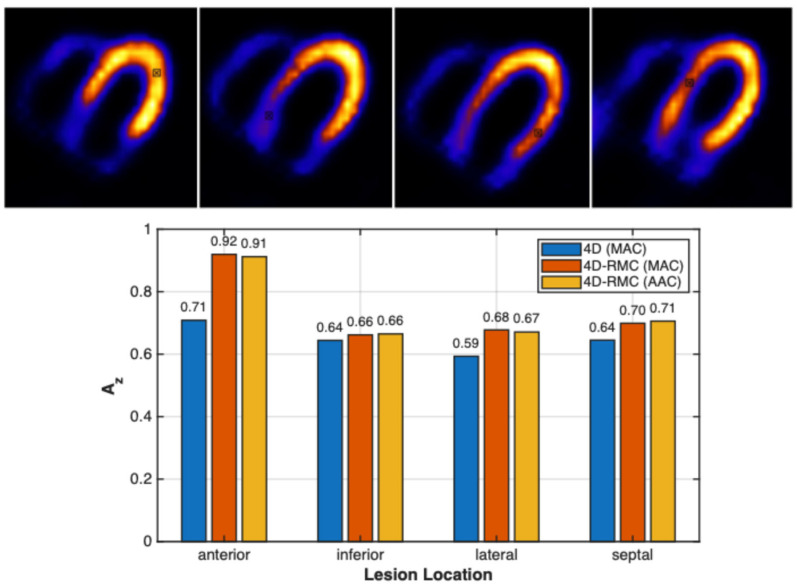
**Top:** the locations (cross in a square) for CHO studies of four lesion cases (on the ideal images); **Bottom:** Az of four lesion cases using different methods.

In Figure 8, we summarize the defect detectability based on the CHO results for four lesion locations (top) using different reconstruction and attenuation correction methods. The CHO results are given by the area under the ROC curve (Az). A higher Az value corresponds to better detection performance. From Figure 8 (bottom), the Az values are significantly higher for 4D-RMC (MAC) and 4D-RMC (AAC) than for 4D (MAC). The maximum improvement of 29.8% is in the anterior location, which suggests that the defect detectability may depend on the lesion location in addition to the reconstruction methods. However, the difference between the 4D-RMC (MAC) and 4D-RMC (AAC) is small for all lesion cases.

The bootstrap-based ROC analysis across four lesion cases showed no statistically significant difference in A_z_ between the 4D-RMC (MAC) and 4D-RMC (AAC) (*p* > 0.05), indicating comparable lesion detectability performance. However, 4D-RMC (MAC) demonstrated a statistically significant improvement over the 4D (MAC) in anterior and lateral cases (both *p* = 0.022), while the other two cases showed no significant differences. These results suggest that the lesion detection performance difference between 4D-RMC and 4D is likely location dependent.

### 4.4. Reconstructed Images

**Figure 9:**
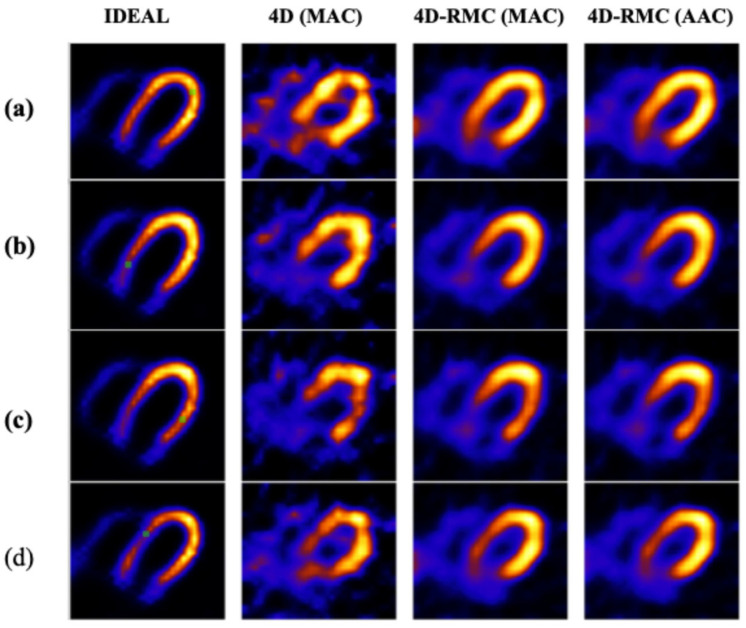
Reconstructed transverse images of left ventricular myocardium for different methods with a lesion located in the (**a**) anterior, (**b**) inferior, (**c**) lateral, and (**d**) septal regions. The green markers denote the lesion locations, and the ideal images serve as a reference.

**Figure 10:**
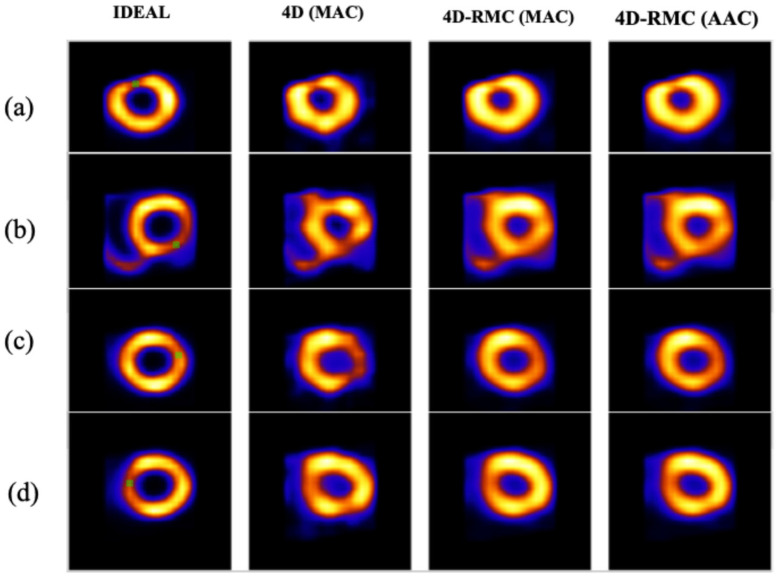
Reconstructed short-axis images of left ventricular myocardium for different methods with a lesion located in the (**a**) anterior, (**b**) inferior, (**c**) lateral, and (**d**) septal regions. The green markers denote the lesion locations and the ideal images serve as a reference.

In Figure 9 and Figure 10, we present a set of transverse and short-axis reconstructed images, respectively, for four lesion locations using different reconstruction and AC methods for a typical noise realization.

From these figures, we observe that 4D (MAC) suffers from high noise which causes distortion of the LV wall, particularly in the inferior and anterior regions. Such artifacts are greatly improved in 4D-RMC images. Furthermore, the LV wall is more uniform in respiratory motion-corrected images in both 4D-RMC (MAC) and 4D-RMC (AAC).

## 5. Discussions

**Figure 11:**
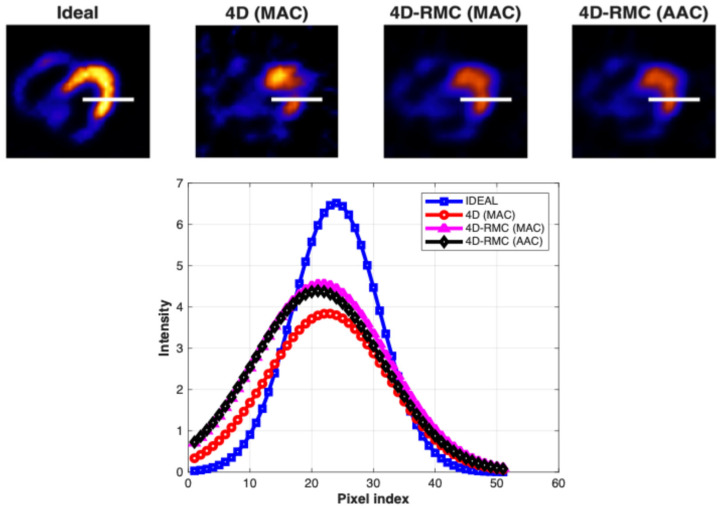
Intensity profiles across the LV wall (off the lesion’s location) in reconstructed transverse slices for different methods for the anterior lesion case.

One of the important aspects of this work is to quantitatively assess whether 4D-RMC (MAC) yields more accurate reconstructions and better lesion detection performance than 4D-RMC (AAC). The results show that 4D-RMC (MAC) did a notably better job in terms of relative RMSE and SSIM than 4D-RMC (AAC). However, the visual inspection of the critical slices and the CHO results do not show significant improvements in 4D-RMC (MAC) over 4D-RMC (AAC). Nevertheless, we found some large improvements in intensity profiles in off-lesion slices. Such a slice in the anterior lesion case is shown in Figure 11. This is consistent with a previous study [28], where respiration-induced misalignment between attenuation and emission maps could introduce quantitative bias even when global image metrics such as relative RMSE and SSIM appear similar, underscoring the importance of motion-matched attenuation correction for reliable regional quantification. This work further demonstrated that the respiratory motion compensation in DG-SPECT greatly improved the reconstruction images over CG-SPECT reconstruction, even with the motion-matched attenuation correction. Although we did not adapt to the fully joint reconstruction, post-4D respiratory motion-compensated filtering showed a close CHO performance to such a complicated fully joint reconstruction [20,29].

While these findings highlight the quantitative potential of the 4D-MAC technique, several modeling simplifications may have limited the observed improvement margin. One such factor is the use of a regular cyclic respiratory motion pattern in obtaining attenuation and activity data using the NCAT phantom. The NCAT model assumes idealized periodic breathing, which fails to capture the complex and irregular respiratory motion typical of clinical populations. In agreement with [30], simplified cyclic motion models may fail to represent the true complexity and variability of patient breathing, potentially limiting the generalizability of motion-corrected reconstruction methods based on idealized motion assumptions.

Another factor is that the 4D NCAT phantom used in this study lacks sufficient diversity from the perspective of anatomical variations due to patient size and population, complex irregular respiratory motion, and breathing patterns. The different body size and motion magnitude of the phantom may lead to different improvement margins. The newer XCAT phantom [31] provides greater physiological realism in these aspects, enabling more accurate evaluation of imaging system performance and motion compensation techniques. We adopted the NCAT phantom over the more versatile XCAT phantom [32] due to the more uniform heart wall of the former. Further comprehensive investigation of the MAC-based 4D respiratory motion compensation (4D-RMC) strategy in clinical settings may be needed for evaluation if it improves other related reconstruction parameters. In the future, simulation studies using the XCAT phantom program could better capture inter-patient variability in anatomies to assess the robustness of the MAC strategies. Consistent with [6], patient-specific and irregular cardiac–respiratory motions remain critical limitations for quantitative accuracy in myocardial perfusion imaging, underscoring the need for advanced correction strategies and motion compensation techniques to ensure reproducible clinical performance.

In addition, for respiratory motion compensation, we used only post-reconstruction temporal smoothing rather than employing joint respiratory motion-compensated reconstruction, the latter of which could potentially achieve an improvement in reconstruction accuracy when combined with motion-matched attenuation correction [20,28]. According to [15], integrating both cardiac deformation and respiratory motion within reconstruction frameworks has been shown to enhance myocardial wall delineation and reduce quantitative inconsistencies, underscoring the value of fully 4D modeling in improving physiological accuracy. Nevertheless, it is also worth noting that the use of motion-compensated temporal smoothing can be more effective in improving image quality than corrections for other degrading factors, such as attenuation [25]. In DG-SPECT, the small misalignment errors in AC may be submerged by the noise suppression from the 4D-RMC framework.

Recent advances in artificial intelligence have demonstrated remarkable potential in SPECT and PET imaging for attenuation map generation, denoising, and end-to-end reconstruction, providing physics-informed compensation for conventional limitations [33,34,35,36,37,38]. Developing deep learning-based motion-compensated reconstruction algorithms capable of tracking complex, irregular respiratory motion—rather than relying on simplified cyclic models—could potentially enhance the performance of motion-matched attenuation correction. Deep learning-based attenuation correction and denoising approaches have also achieved attenuation map generation without additional CT exposure while enhancing image quality and quantitative reliability [39,40]. Notably, projection-domain conditional generative adversarial network (cGAN)-based denoising enables significant dose reduction without compromising diagnostic accuracy [33,39]. Integrating these data-driven models with physics-based 4D reconstructions could enable hybrid frameworks that jointly address attenuation, scatter, and motion in a unified, learning-driven paradigm, which would be a future research direction.

Building on these algorithmic advances, the translation into clinical workflows requires careful consideration of gating and synchronization constraints. As shown in [17], even minor misregistration between SPECT and CT images can introduce notable quantitative bias in reconstructed activity and uptake estimates, underscoring the importance of the accurate respiratory motion-matched attenuation correction for DG-SPECT. Similar technical challenges in dual gating and data-driven synchronization have also been noted by [41]. In this study, we used respiratory phase-synchronized attenuation correction (AC) maps during respiratory motion compensation in DG-SPECT. Although dual cardiac-respiratory AC could further improve alignment between attenuation and activity maps, acquiring dual-gated attenuation maps would require CT synchronization with both respiratory and ECG signals, substantially increasing data-processing complexity. Moreover, in real clinical settings, cardiac and respiratory irregularities are common, posing additional challenges for motion-matched AC. Nevertheless, mitigating these mismatched AC artifacts is expected to yield greater quantitative and diagnostic benefits. To enable clinical implementation of dual cardiac-respiratory gating, extensive testing using phantoms and patient datasets encompassing diverse anatomical and physiological characteristics will be essential. Overall, this study establishes a foundation for incorporating motion-matched attenuation correction into 4D reconstruction workflows and highlights the need for clinically adaptive models, such as deep learning driven models, to handle complex and irregular motions.

## 6. Conclusions

In this work, we quantitatively evaluated two attenuation correction approaches—motion-matched attenuation correction (MAC) and motion-averaged attenuation correction (AAC)—within a respiratory motion-compensated four-dimensional reconstruction (4D-RMC) framework for dual cardiac–respiratory gated (DG) SPECT. The results demonstrated that 4D-RMC effectively suppresses elevated noise in DG-SPECT, yielding improved reconstruction quality compared with conventional cardiac-gated (CG) SPECT. Although MAC produced slightly better quantitative performance in terms of relative RMSE and SSIM, its impact on lesion detectability and overall image quality remained modest, likely due to the use of an idealized cyclic motion model and limited anatomical variability in the NCAT phantom.

These findings underscore that while 4D motion compensation and attenuation matching can enhance quantitative accuracy, the full clinical potential of MAC may require more diverse phantoms, incorporating irregular respiratory motion and anatomical variability, as well as advanced modeling frameworks. Further investigation leveraging versatile XCAT-based simulations and deep learning-driven reconstruction and compensation strategies capable of tracking complex motion patterns could provide stronger evidence for the clinical benefit of motion-matched attenuation correction in DG-SPECT MPI.

## Data Availability

The data and code can be accessed at https://github.com/noor-rasel/DG_SPECT.
